# New Insights into the Dermocosmetic Potential of the Red Seaweed *Gelidium corneum*

**DOI:** 10.3390/antiox12091684

**Published:** 2023-08-29

**Authors:** Margarida Matias, Alice Martins, Celso Alves, Joana Silva, Susete Pinteus, Manuel Fitas, Pedro Pinto, Joana Marto, Helena Ribeiro, Patrick Murray, Rui Pedrosa

**Affiliations:** 1 MARE-Marine and Environmental Sciences Centre and ARNET-Aquatic Research Network, Escola Superior de Turismo e Tecnologia do Mar, Polytechnic of Leiria, 2520-630 Peniche, Portugal; celso.alves@ipleiria.pt (C.A.); joana.m.silva@ipleiria.pt (J.S.); susete.pinteus@ipleiria.pt (S.P.); rui.pedrosa@ipleiria.pt (R.P.); 2 LIFE-Health and Bioscience Research Institute, Technological University of Shannon, Moylish Park, V94 E8YF Limerick, Ireland; patrick.murray@tus.ie; 3 PhD Trials, Avenida Maria Helena Vieira da Silva, n° 24 A, 1750-182 Lisboa, Portugal; mfitas@phdtrials.com (M.F.); pfcpinto@ff.ulisboa.pt (P.P.); 4 Research Institute for Medicines (iMed.ULisboa), Faculty of Pharmacy, Universidade de Lisboa, 1649-003 Lisbon, Portugal; jmmarto@ff.ulisboa.pt (J.M.); hribeiro@campus.ul.pt (H.R.)

**Keywords:** *Gelidium corneum*, greener skincare products, marine natural products, antioxidant capacity, photoprotection, wound healing, topical formulations, in vivo compatibility

## Abstract

This work addresses the potential of the red seaweed *Gelidium corneum* as a source of bioactive ingredients for skin health and wellness in response to the growing awareness regarding the significance of sustainable strategies in developing new nature-based dermocosmetic products. Hydroalcoholic extracts from the dried biomass were subjected to sequential liquid–liquid partitions, affording five different fractions (F1–F5). Their cosmetic potential was assessed through a set of in vitro assays concerning their antioxidant, photoprotective, and healing properties. Additionally, their cytotoxicity in HaCaT cells and their capacity to induce inflammation in RAW 264.7 cells were also evaluated. As a proof-of-concept, O/W emulsions were prepared, and emulsion stability was assessed by optical microscopy, droplet size analysis, centrifugation tests, and rheology analysis. Furthermore, in vivo tests were conducted with the final formulation to assess its antioxidant capacity. At subtoxic concentrations, the most lipophilic fraction has provided photoprotection against UV light-induced photooxidation in HaCaT cells. This was conducted together with the aqueous fraction, which also displayed healing capacities. Regarding the physical and stability assays, the best performance was achieved with the formulation containing 1% aqueous extract, which exhibited water retention and antioxidant properties in the in vivo assay. In summary, *Gelidium corneum* displayed itself as a potential source of bioactive ingredients with multitarget properties for dermatological use.

## 1. Introduction

The cosmetic industry remains a major focus of economic development in the 21st century, being actively interlocked with health-based concerns and environmental awareness. Issues such as biodegradability, sustainable ingredient sources, and fair labor are prevalent in communities with a high literacy percentage and should not be disregarded [1,2].

Molecules from natural sources offer some unique benefits, boosting consumers’ confidence in products and showing great potential compared to chemically synthesized ones. Due to consumers’ demands and the health benefits shown by these compounds, there has been a reported increase in their inclusion in cosmetic formulations [3,4].

Skin is the human body’s external barrier and its main shield against the sun. Ultraviolet (UV) radiation increases the production of reactive oxygen species (ROS), which can result in an oxidative stress condition by activating the arachidonic acid pathway and increasing inflammatory responses [5]. In addition, acute UV radiation can cause sunburn and hyperpigmentation, while long-term exposure can lead to the development of skin cancer [6]. Oxidative stress is a result of ROS accumulation, and this condition can lead to cellular senescence by causing lipid, protein, nucleic acid, and organelle damage. Skin UV damage not only increases ROS production but also enables inflammation and irritation in epidermal cells [7,8]. Combating this effect is important to ensure a healthy barrier. Therefore, antioxidant components are highly valuable in cosmetics, offering not only UV protection but also helping to diminish tissue inflammation due to the deterrence of ROS occurrence and prevention of the oxidative condition [9,10].

The marine environment is characterized by incredible biodiversity as well as the occurrence of multiple stress factors that have an impact on species’ survival. As a defense mechanism, those organisms need to activate secondary metabolic pathways to produce bioactive molecules with unique structural features that can be profited by the pharmaceutical, feed, food, and cosmetic sectors.

Seaweeds offer a wide range of possibilities for the extraction of biomolecules of interest [11]. Macroalgae thrive in the intertidal zone, and this challenging environment requires a huge deal of physiological plasticity to handle stress. Organisms living in this area must adapt and develop defense mechanisms to handle repeated biotic and abiotic fluctuations, especially if they possess no form of mobility [12]. These mechanisms give way to the synthesis of biomolecules such as terpenoids, alkaloids, polyketides, peptides, steroids, phenolics, polysaccharides, lipids, and pigments, among others [13]. Some of them have already been applied in the cosmetic industry [14], given their beneficial effects on skin health. In particular, mycosporine-like amino acids (MAAs) biosynthesized by red seaweeds can confer photoprotection due to their capacity to absorb ultraviolet radiation or reduce the impact of free radicals on cells [15,16,17].

*Gelidium corneum* is a red seaweed commonly found on the Portuguese shore that is almost exclusively harvested as a source of agar. However, other interesting biomolecules, including MAAs, phycobiliproteins, sulfated polysaccharides, etc., could be extracted from this marine resource [16,18,19,20]. Therefore, the aim of this study was to assess the potential of *G. corneum* collected in Portugal coast as a source of ingredients with antioxidant, photoprotective, and healing effects. This study was looking to achieve the valorization of this marine resource through the development of novel and sustainable natural cosmetic formulations.

## 2. Materials and Methods

### 2.1. Seaweed Harvest and Sampling

*Gelidium corneum* (Lamouroux, 1813) was collected in October 2020 at Praia dos Barcos (39°22′35.9″ N 9°20′23.7″ W), Peniche, Portugal, and immediately transported to the laboratory facilities of MARE-Polytechnic of Leiria. Seaweed biomass was washed with seawater to remove invertebrate organisms and debris, and posteriorly it was weighed and dried at 60 °C in an oven with air circulation. Then, dried samples were ground in a food mill (Moulinex, Paris, France) and stored in a dry, dark place until extraction procedures.

### 2.2. Extraction and UV–Vis Characterization of Extracts

**Figure 1:**
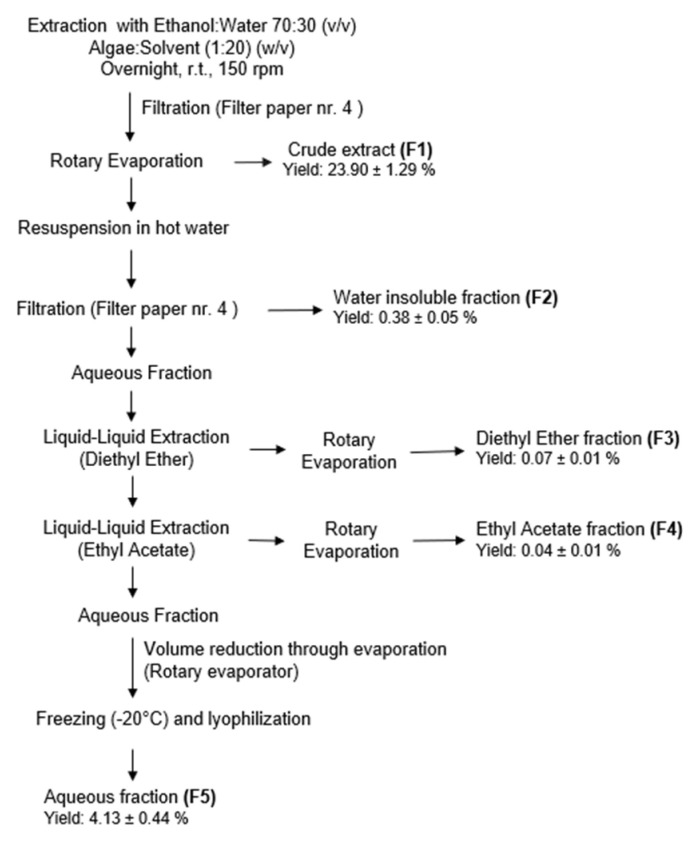
*Gelidium corneum* extraction and fractionation steps.

The fractionation process was performed as previously described [21]. Solvents *p.a.* (VWR-BDH Chemicals, Fontenay-sous-Bois, France) were selected according to EU Regulation No. 1223/2009 for cosmetic application. For each extraction, 100 g of powdered seaweed were weighed. Extractions (ethanol:water, 70:30) were performed at room temperature (20 ± 2 °C) under constant stirring (150 rpm) over 17 h, protected from light. Extracts were filtered on qualitative filter paper (FP) nr. 4 (VWR International, Alcabideche, Portugal) and concentrated under vacuum at a low temperature (40 °C) in a rotary evaporator (IKA HB10, VWR International, Alcabideche, Portugal). The dried crude extract (F1) was resuspended in ultrapure water (Advantage A10 Milli-Q lab, Merck, Darmstadt, Germany) previously warmed to 75–80 °C and filtered (FP nr. 4), affording a solid, insoluble fraction (F2) and an aqueous fraction. After cooling to room temperature, this last one was subjected to a liquid–liquid partition, firstly with diethyl ether (F3) and then with ethyl acetate (F4), which were concentrated to dryness in a rotary evaporator. The remaining aqueous fraction (F5) was also evaporated until half volume was obtained, then frozen (−20 °C), and lyophilized. The extraction/fractionation scheme is represented in Figure 1.

The fractions (F1, F2, F3, F4, and F5) were analyzed in a UV–visible spectrophotometer (Evolution 201, Perkin Elmer, Waltham, MA, USA) with a spectral window from 200 to 800 nm.

### 2.3. Evaluation of Gelidium corneum’s Biological Properties

For in vitro bioassays, samples and controls were dissolved in water and/or dimethyl sulfoxide (DMSO) at 100 mg/mL.

#### 2.3.1. Antioxidant Capacity Assessment 

##### Total Phenolic Content 

The total phenolic content (TPC) of fractions was determined using the Folin–Ciocalteu method adapted to the microplate scale as described by Pinteus et al. [22]. Gallic acid (GA) was used as a standard for the calibration curve, following the same steps. TPC is expressed as milligrams of gallic acid equivalents per gram of dry extract (mg GAE/g of extract).

##### DPPH Radical Scavenging Activity

The DPPH radical scavenging activity was assessed according to Silva et al. [23]. For fractions that reduce more than 50% of the available DPPH radical at a maximum concentration of 1000 µg/mL, EC_50_ values (μg/mL) were determined. Several concentrations (0–1000 μg/mL) of each fraction were tested.

##### Ferric Reducing Antioxidant Power (FRAP)

The FRAP reagent was prepared with 300 mM acetate buffer (pH 3.6), 10 mM of 2,4,6-Tri(2-pyridyl)-*s*-triazine (TPTZ) in 40 mM HCl, and a 20 mM ferric solution using FeCl_3_. By freshly mixing acetate buffer, TPTZ, and ferric solutions at a ratio of 10:1:1, the final working FRAP reagent was incubated at 37 °C. Then, 2 μL of the sample were added to 198 μL of FRAP reagent, and the reaction remained in the dark for 30 min. After incubation time, the absorbance was measured at 593 nm in the microplate reader (Epoch, BioTek^®^ Instruments, Winooski, VT, USA). FeSO_4_ was used as a standard for the calibration curve. The difference between the absorbance of the samples and the blank was calculated and expressed as μM of FeSO_4_ equivalents per gram of dry extract (μM FeSO_4_/g extract).

##### Oxygen Radical Absorbance Capacity (ORAC)

The ORAC assay was carried out as described by Dávalos et al. [24]. The reaction was carried out in phosphate buffer (75 mM, pH 7.4), and the final reaction volume was 200 µL. In a 96-well microplate, 20 µL of each sample and 120 µL of fluorescein (Sigma-Aldrich, Steinheim, Germany) (70 mM) were added. The mixture was pre-incubated at 37 °C for 15 min. After this period, 60 µL of the 2,2′-Azobis(2-methylpropionamidine) dihydrochloride (AAPH) solution (Sigma-Aldrich, Steinheim, Germany) were added. The plate was placed in the microplate reader (Multimodal Synergy H1, BioTek^®^ Instruments, Winooski, VT, USA), and fluorescence (excitation: 458 nm; emission: 520 nm) was recorded every minute for 180 min and automatically shaken before each reading. Eight calibration solutions were prepared using Trolox (Merck, Darmstadt, Germany) (0–80 μM) as an antioxidant standard; a blank was also conducted using phosphate buffer instead of fluorescein. ORAC values were expressed in μmol of Trolox equivalents/g extract (μmol TE/g extract).

#### 2.3.2. Evaluation of Biological Activities in In Vitro Cellular Models 

##### Cell Culture Maintenance

Human keratinocytes (HaCaT cells—300493) were acquired from Cell Lines Services, Germany (CLS). HaCaT cells were cultured in Dulbecco’s Modified Eagle’s Medium (DMEM) high glucose medium supplemented with 10% fetal bovine serum (FBS) and 1% antibiotic/antimycotic solution (amphotericin B, 0.25 mM; penicillin, 60 mM; streptomycin, 100 mM). The subculture of HaCaT cells was accomplished after the cells reached total confluence using trypsin-EDTA to dissociate the cells. Murine macrophages RAW 264.7 (ATCC: TIB 71) were acquired from the American Type Culture Collection and cultured in DMEM supplemented with 4.5 g glucose, 0.35 g/L l-glutamine, 10% FBS, 1% antibiotic/antimycotic solution, and 1% sodium pyruvate. Both cell lines were cultivated at 37 °C with 5% CO_2_ and 95% humidity. Subculture was performed according to biobank instructions whenever cultures reached 80–85% confluence.

##### Cytotoxicity 

The cytotoxic activity of *G. corneum* fractions was evaluated using a 3-[4, 5-dimethylthiazol-2-yl]-2, 5-diphenyltetrazolium bromide (MTT) colorimetric assay, according to Yuan et al. [25] with slight modifications. After seeding in 96-well plates and incubation overnight, HaCaT cells (4 × 10^4^ cells/well) and RAW 264.7 cells (5 × 10^4^ cells/well) were treated with the fractions at the maximum concentration of 1000 µg/mL for 24 h. Then, the culture medium was removed, and 100 μL of MTT (0.5 mg/mL) were added to the cells, followed by an incubation time of 30 min in the dark at the regular culture conditions mentioned above. The intracellular formazan crystals were solubilized with 100 μL DMSO. After 24 h, the absorbance was measured at 570 nm using a microplate reader (Epoch Microplate Spectrophotometer, BioTek, Winooski, VT, USA). The results were expressed as a percentage of untreated control cells.

##### Photoprotective Capacity

The photoprotective effect of fractions was determined according to Marto et al. [26], with slight modifications. This assay was evaluated through ROS production measurements on HaCaT cells exposed to ultraviolet radiation (UVA and UVB) using the fluorophore 2′,7′-dichlorodihydrofluorescein diacetate (H_2_DCFDA) (Thermo FischerScientific, Eugene, OR, USA). HaCaT sub-confluent cells were grown in 96-well plates and incubated in the dark for 30 min with H_2_DCFDA (20 µM) at 37 °C. The medium was then removed, and fresh medium was added to the cells before they were treated with the fractions at non-cytotoxic concentrations (100–1000 μg/mL) for 1 h. *N*-acetyl cysteine (NAC) was used as a positive control at 10 µM. Subsequently, the medium was removed, and the cells were washed with fresh medium and then exposed to UVA–B radiation using a simulator (UVA Cube 400, Hönle Technology, Gräfelfing, Germany) with an intensity of 7.9 mW/cm^2^ for 15 min. Cells were irradiated with a UV lamp equipped with an H2 filter (UVA + UVB) with a spectral output of 280–400 nm, and the intensity of UV radiation was measured using a sensor (UV-Metro, Hönle Technology, Gräfelfing, Germany). Then, ROS levels were determined by fluorescence measurement (Multimodal Synergy H1, BioTek^®^ Instruments, Winooski, VT, USA) at an excitation of 495 nm and emission of 527 nm wavelengths recorded each minute for 10 min. The results were expressed as a percentage of the negative control, which contained only the vehicle at the same concentrations as the fractions.

##### Wound Healing Assay 

To measure possible effects on wound healing capacity, a scratching and healing assay was performed on HaCaT cells. Firstly, the cells were seeded into a 96-well plate and left there until confluence was achieved. Then, the individual wells were scratched with a 10 μL pipette tip, splitting the cells, which were then depleted of medium and washed twice with 100 μL of PBS. Afterward, 100 µL of medium was added, and images were taken at 100× using a camera inserted in a fluorescence inverted microscope (ZEISS Axio, VERT. A1, equipped with an AxioCam MRC-ZEISS camera, Oberkochen, Germany). The culture medium was removed and replaced with a medium containing non-cytotoxic concentrations of seaweed fractions (100–1000 μg/mL). After 12 h (a short incubation time that would not allow extensive cell proliferation), the images were taken once again. The results are presented as a percentage of the total healed area.

##### Inflammatory Effect 

To assess safety for human volunteers, the inflammatory effect of *G. corneum* fractions was estimated through nitric oxide (NO) production using a methodology adapted from Yang et al. [27]. RAW 264.7 cells were seeded (5 × 10^4^ cells/well) into 96-well plates and grown overnight. Cells were then incubated with non-toxic concentrations of seaweed fractions for 24 h. To measure NO production, 150 μL of cellular medium were transferred into a new plate, and 50 μL of previously prepared Griess reagent (1% (*w*/*v*) sulphanilamide, 0.1% (*p*/*v*) *N*-(1-naphtyl) ethylenediamine, and 2.5% (*v*/*v*) phosphoric acid) were added. After 30 min of incubation in the dark at room temperature, the absorbance was measured at 546 nm using a microplate reader (Epoch, BioTek^®^ Instruments, Winooski, VT, EUA). Results are presented in relation to the control untreated cells (%). 

### 2.4. Development and Characterization of a Topical Formulation Containing Gelidium corneum Aqueous Extract

#### 2.4.1. Ingredients

Liquid paraffin, almond oil, and glycerin were obtained from A. M. S. Cruz, Material de Laboratório, Lda. (Lisbon, Portugal). Polyglyceryl-3 dicitrate/stearate (Tego Care PSC3^®^), cetearyl alcohol (Tego Alkanol 1618^®^), and decyl oleate (Tegosoft DO^®^) were obtained from Evonik Industries AG (Essen, Germany). Methylparaben (Nipagin^®^) and propylparaben (Nipasol^®^) were obtained from Fagron Iberica S.A.U. (Barcelona, Spain). Purified water was obtained by reverse osmosis (Millipore, Elix 3, Millipore Corporation, Billerica, MA, USA).

#### 2.4.2. Preparation of the Emulsion

Formulations were prepared according to Marques et al. [28], as described in the following: Three O/W emulsions were prepared using two different extract concentrations (emulsion 1% and emulsion 10%), as well as a control blank consisting of cream without any added extract. The 10% extract concentration was used as the worst-case scenario regarding the impact of these extracts on the structure of the formulations. The composition of the formulations is shown in Table 1. 

To prepare the emulsions, the oily and aqueous phases were heated separately to 75 °C. Then, the oily phase was added to the water phase, and the system was mixed at 150 rpm with constant agitation until 30 °C. The pH was confirmed to be between 5.5 and 6 with a pH-Meter, SevenEasy™ (Mettler Toledo, Greifensee, Switzerland), at room temperature. The extracts were mixed into the formulation, and the emulsions were packed into the container.

### 2.5. Formulation Stability Assessment 

To further assess the effect of extract concentration on the stability of the prepared emulsions, different tests were performed after 7 days of storage. Organoleptic characteristics of formulations, such as appearance, color, odor, and pH, were evaluated.

#### 2.5.1. Macroscopic Analysis and Centrifugation Test 

The macroscopic appearance of each formulation was visually analyzed and used as the first stability indicator. The second parameter to evaluate concerning stability was the behavior of samples under centrifugation (Medifuge, Heraeus Sepatech GmbH, Hanau, Germany) at 4000 rpm during a 15 min cycle. 

#### 2.5.2. Droplet Size Analysis

The emulsions were analyzed for droplet size using an optical microscope with polarized light (Nikon Eclipse Ci, Nikon, Tokyo, Japan) incorporated with a camera (Sony Exmor CMOS Sensor, MicroCopiaDigital, Münster, Germany). The droplet size distribution of the emulsions was measured by light scattering using a Malvern Mastersizer 2000 (Malvern Instruments, Worcestershire, UK) with a Hydro 2000S(A) accessory. For correct turbidity, about 0.5 g of each formulation, corresponding to an obscuration between 15% and 16%, was added to the sample chamber containing 150 mL of water using a stirrer at 1750 rpm. Data are expressed in terms of the relative distribution of the volume of particles in the range of size classes. 

#### 2.5.3. Rheology Studies

The emulsions with *G. corneum* and the blank were analyzed concerning their consistency, according to Nunes et al. [29]. Briefly, the rheological characteristics were measured with a controlled stress Kinexus Lab+ Rheometer (Malvern Instruments, Worcestershire, UK) using cone–plate geometry (truncated cone angle of 4° and radius of 40 mm). All the studies were performed at 25 °C. The shear stress of each emulsion was obtained by increasing the shear rate from 0.1 s^−1^ to 100 s^−1^. Regarding the oscillatory method, a frequency sweep test was performed with a shear strain of 0.1% and a frequency range between 0.1 and 10 Hz.

### 2.6. In Vivo Compatibility, Acceptability, and Antioxidant Efficacy

This study aimed to check the formulations’ compatibility and acceptability and assess their antioxidant efficacy. The cutaneous acceptability and compatibility studies were supervised by a dermatologist, who evaluated the results after a visual examination of the experimental area and questioning of the subjects. The antioxidant efficacy of the topical formulations was evaluated by the in vivo assay, as described by Marques et al. [28]. Briefly, the skin color was determined using a tristimulus color analyzer that measures the reflected color. A Chromameter CR-400^®^ (Minolta, Tokyo, Japan) was used to detect any slight deviation in the xenon’s light spectral distribution. The system provides data for the luminance (L*), a* (red–green), and b* (blue–yellow) color distributions. For the antioxidant efficacy assay, 10 healthy volunteers aged 18–65 years were chosen. All volunteers were informed about the procedure and signed an informed consent form. The same specific criteria for non-inclusion of the test were applied. The study code is 20250222.C, E, and it was accomplished on 20 June 2022. This is a single-center, blind-controlled study in healthy subjects. The protocol was sufficient to check acceptability and assess the product’s efficacy.

### 2.7. Statistical Analysis

To determine possible significant differences relative to the control, a one-way analysis of variance (ANOVA) with Dunnett’s multiple comparison tests was used. All data were checked for normality (Shapiro–Wilk test) and homoscedasticity (Levene test). When requirements for an ANOVA were not met, a non-parametric Kruskal–Wallis and Dunn’s multiple comparison test was applied. The IC_50_ and EC_50_ values were determined using the GraphPad v9.3.1 software through the equation y = 100/(1 + 10 (X − Log IC_50_)). Calculations and final graphical representations were performed using GraphPad v9.3.1 (GraphPad Software, La Jolla, CA, USA). All data were obtained from at least three independent experiments and are presented as standard deviation (SD) or mean ± standard error of the mean (SEM) with a significance level of 0.05 (*p* < 0.05).

Regarding in vivo assays, a comparative analysis was performed by the Wilcoxon signed-rank test for paired data (non-parametric) if the distribution was not normal. A paired sample Student’s *t*-test was used for paired data if the distribution was normal. In both cases, the significance level adopted was 95%. All the calculations were performed using SPSS, version 22 (IBM). The subjective data of efficacy were submitted to a suitable statistical treatment, the Binomial test and a Chi-squared test.

## 3. Results

### 3.1. Extraction and UV–Vis Absorption Spectra of Extracts 

The best extraction yield was achieved with the hydroalcoholic crude extract F1 (23.90 ± 1.29%), followed by the aqueous fraction F5 (4.13 ± 0.44%). The water-insoluble fraction F2 (0.38 ± 0.05%), the diethyl ether F3 (0.07 ± 0.01%), and the ethyl acetate F4 (0.04 ± 0.01%) fractions exhibited lower extraction yields.

**Figure 2:**
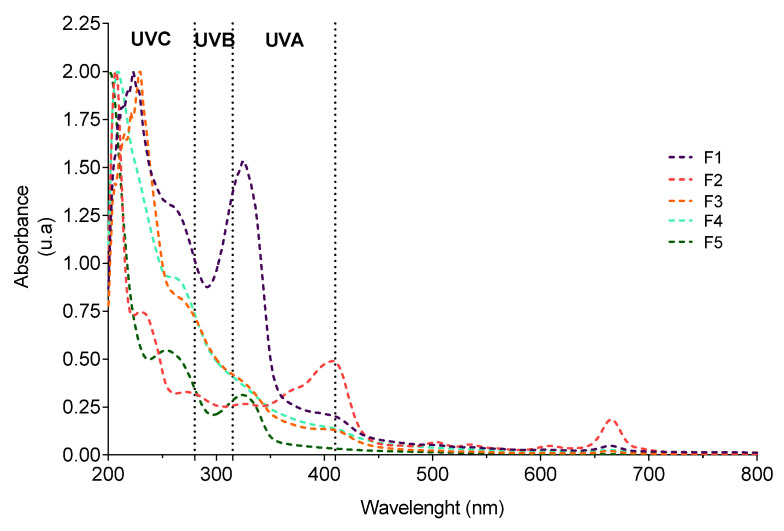
UV–visible absorption spectra (200–800 nm) of *Gelidium corneum* fractions.

The UV–Vis absorption spectra of all fractions are depicted in Figure 2.

Overall, *G. corneum* fractions exhibited absorption maximums in the UVA (410–315 nm), UVB (315–280 nm), and UVC (280–200 nm) regions (Figure 2). Additionally, absorption peaks in the visible region (420–680 nm) characteristic of pigments such as chlorophylls and carotenoids were also observed in the F1, F2, F3, and F4 fractions. Absorption maxima at 260 nm and 326 nm were also displayed by the most polar fractions (F1 and F5), which can be attributed to MAAs [16], suggesting the presence of such a group of compounds in both fractions. This is also supported by proton signals between 2.5 and 3.5 ppm, characteristic of MAAs [16], observed in a previous analysis by NMR [21]. 

### 3.2. Antioxidant Capacity

The total phenolic content (TPC) and the antioxidant capacity of each fraction (F1–F5) were evaluated through three different in vitro assays (DPPH, FRAP, and ORAC), and the results are shown in Table 2.

Among the studied samples, the ethyl acetate fraction (F4) displayed the highest total phenolic content (32.92 ± 6.29 mg GAE/g extract), followed by F3 (12.80 ± 2.28 mg GAE/g extract), F2 (10.64 ± 1.19 mg GAE/g extract), F1 (4.37 ± 1.54 mg GAE/g extract), and F5 (3.63 ± 0.29 mg GAE/g extract). 

Fraction F3 exhibited the best capacity to reduce the DPPH radical, while fractions F1 and F5 did not reduce DPPH by more than 50%. Dose-dependent assays were performed to calculate the EC_50_ value. The fraction with the highest capacity to reduce the DPPH radical was F3, with a notably smaller EC_50_ value (399.60 μg/mL) compared with F4 (973.10 μg/mL) and F2 (991.60 μg/mL). Nevertheless, none of the selected extracts exhibited a stronger effect than the standard BHT (butylated hydroxytoluene) (EC_50_ = 184.70 μg/mL).

In the FRAP assay, once again, F3 displayed the best result (49.02 ± 5.27 μM FeSO_4_ EQ/g extract). Yet, this result still differs from BHTs’ ability to reduce ferric ions. In the ORAC assay, fractions F2, F3, and F4 highly surpassed BHT antioxidant capacity.

Regarding the ORAC assay, F4, F3, and F2 displayed the best results (3060.00 ± 222.20 μmol TE/g extract, 2916.00 ± 132.80 μmol TE/g extract, and 2868.00 ± 72.29 μmol TE/g extract, respectively). However, the statistical analysis (Kruskal–Wallis, Dunn’s, *p* < 0.05) revealed no significant differences between them, despite having significant differences with the control (BHT-136.4 ± 9.09 μmol TE/g extract).

### 3.3. Gelidium corneum Biological Activities in In Vitro Cellular Models 

#### 3.3.1. Cytotoxicity

**Figure 3:**
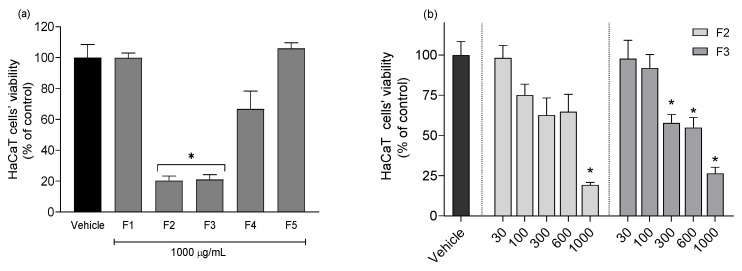
Cytotoxicity of *Gelidium corneum* fractions at (**a**) maximum concentration of 1000 μg/mL and (**b**) dependence of the concentration for the most cytotoxic fractions (300–1000 μg/mL) after 24 h of treatment on HaCaT cells. Values in each column represent the mean ± standard error of the mean (SEM) of three independent experiments carried out in triplicate. The symbol (*) represents significant differences (ANOVA, Dunnett’s test, *p* < 0.05) when compared to the vehicle.

The cytotoxic effect of *G. corneum* fractions on HaCaT cells is displayed in Figure 3.

At the highest concentration (1000 μg/mL), fractions F2 and F3 from *G. corneum* exhibited a marked reduction in cell viability, with only 19.30 ± 1.60% and 26.37 ± 3.84% remaining viable cells, respectively. Fractions F1, F4, and F5 did not show significant differences in relation to the vehicle situation. Due to the results observed at the maximum concentration, different concentrations of F2 and F3 were tested to define the non-cytotoxic concentration (Figure 3b). In the end, fraction F3 was revealed to be the most cytotoxic one, only stopping to affect cell viability at 100 μg/mL.

#### 3.3.2. Inflammatory Effect Assessment

**Figure 4:**
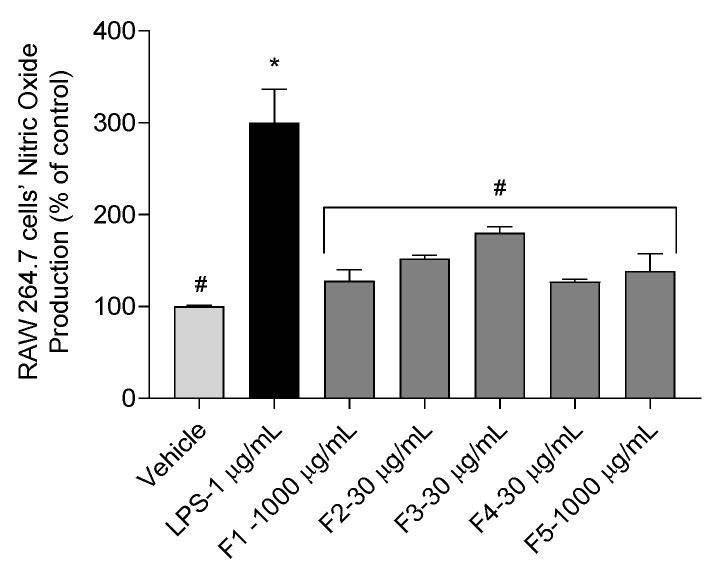
Inflammatory effects of *Gelidium corneum* fractions on RAW 264.7 cells through nitric oxide (NO) production after 24 h of exposure at sub-toxic concentrations (30–1000 μg/mL). Lipopolysaccharide (LPS, 1 μg/mL) was used as a positive inflammation control. Values in each column represent the mean ± SEM of three independent experiments conducted in triplicate. The symbol (*) represents significant differences when compared to the vehicle. Symbol (#) represents significant differences when compared to LPS (ANOVA, Dunnett’s test, *p* < 0.05).

A cosmetic should not induce severe inflammation upon use. Therefore, for safety reasons, it is important to assess if the studied seaweed fractions can still cause inflammation at non-cytotoxic concentrations, which were 1000 μg/mL for F1 and F5 and 30 μg/mL for the remaining fractions. Results are shown in Figure 4. 

The results of this assay show that, at non-cytotoxic concentrations, none of the fractions increase NO production in RAW 264.7 cells when compared with the vehicle (100.00 ± 0.64%).

#### 3.3.3. Photoprotective Effect

**Figure 5:**
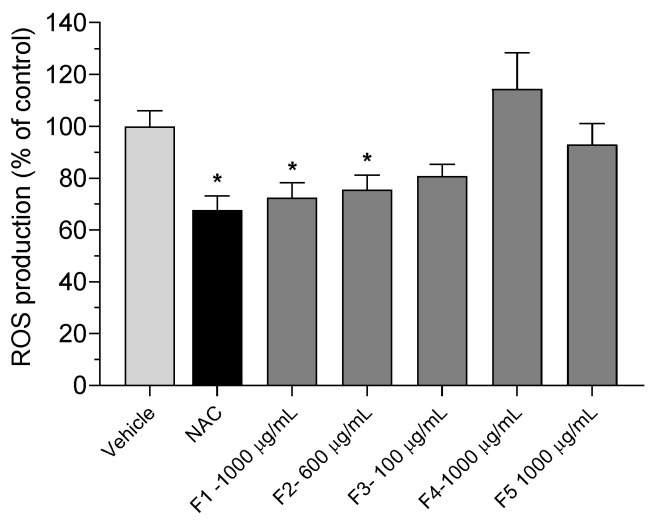
Production of reactive oxygen species by HaCaT cells in the presence of *Gelidium corneum* fractions (100–1000 μg/mL) when exposed to UVA–B radiation (7.9 mW/cm^2^) (15 min). Values in each column represent the mean ± standard error of the mean (SEM) of three independent experiments. Symbols (*) represent significant differences (ANOVA, Dunnett’s test, *p* < 0.05) when compared to the vehicle; no statistical differences between paired fractions were found (ANOVA, Dunn’s test, *p* < 0.05); NAC (10 μg/mL) was used as a positive control.

The photoprotective potential of *G. corneum* fractions on HaCaT cells exposed to UVA–B radiation was evaluated, and results are depicted in Figure 5.

Regarding the photoprotective capacity of this seaweed, only the crude extract F1, at 1000 μg/mL, and the water-insoluble fraction F2, at 600 μg/mL, were able to reduce the ROS production when compared with the vehicle, with F1 displaying the best result with 27.52 ± 5.84%. 

#### 3.3.4. Wound Healing Capacity

**Figure 6:**
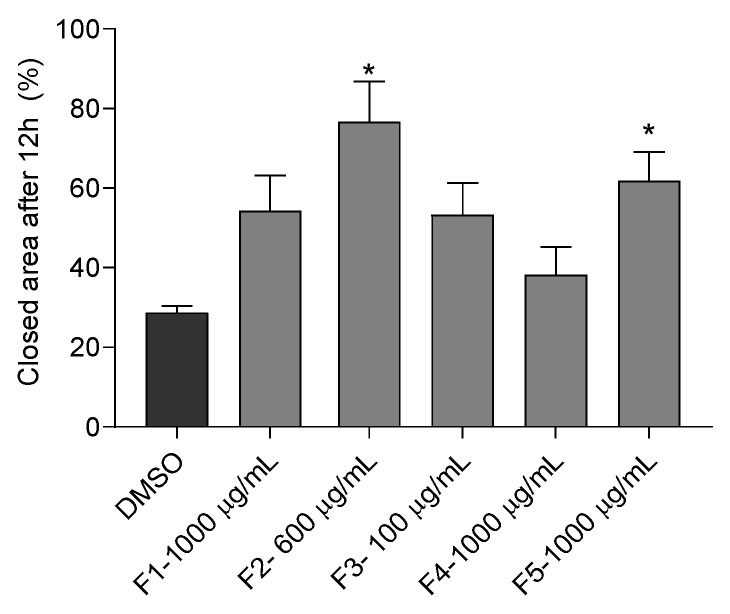
Closed area (%) of *Gelidium corneum* fractions after 12 h. The values in each column represent the mean ± SEM of three independent experiments. The symbol (*) represents significant differences when compared to the vehicle (ANOVA, Dunnett’s test, *p* < 0.05).

The wound healing capacity of *G. corneum* fractions on HaCaT cells was assessed, and the results are depicted in Figure 6.

The healing capacity was noticeable with the F2 (76.76 ± 10.02%) and F5 (61.83 ± 7.25%) fractions. On the other hand, fractions F1, F3, and F4 did not promote cells’ healing.

### 3.4. Cosmetic Topical Formulations 

To choose a suitable fraction for cosmetic incorporation, the in vitro bioactivity results were considered, and the extraction yields and sensorial aspects, such as color, smell, and solubility in water. Fractions F1, F2, F3, and F4 presented a quite vibrant dark green color, paired with a strong smell. Although F5 did not display a particularly high antioxidant potential in the in vitro assays, the results stated in the healing assay, the higher yield and higher solubility in water, as well as the seeming inert effect on some skin microorganisms [21], contributed to choosing this fraction for further cosmetic incorporation.

#### Stability Assessment of Emulsions Formulated with *G. corneum* Aqueous Extract

After the previous assessment, three emulsions were prepared as described above using the aqueous *G. corneum* extract (F5). All emulsions were semisolid, and the blank emulsion was white. In contrast, the emulsions containing the extracts (1% and 10%) presented a caramel tone. After 7 days of storage, only the formulations with a higher percentage of extract were more consistent, presenting phase separation after a centrifugation test. 

##### Droplet Size Analysis

**Figure 7:**
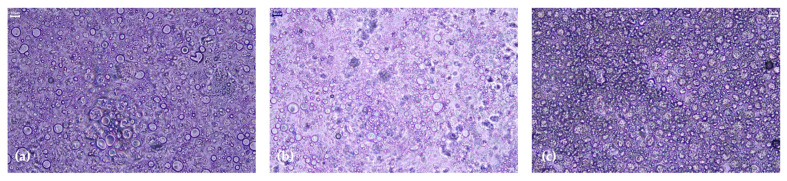
Photographs of different emulsions with *Gelidium corneum* (F5) under the microscope at 200× (**a**) Blank, (**b**) 1% Extract, and (**c**) 10% Extract.

**Figure 8:**
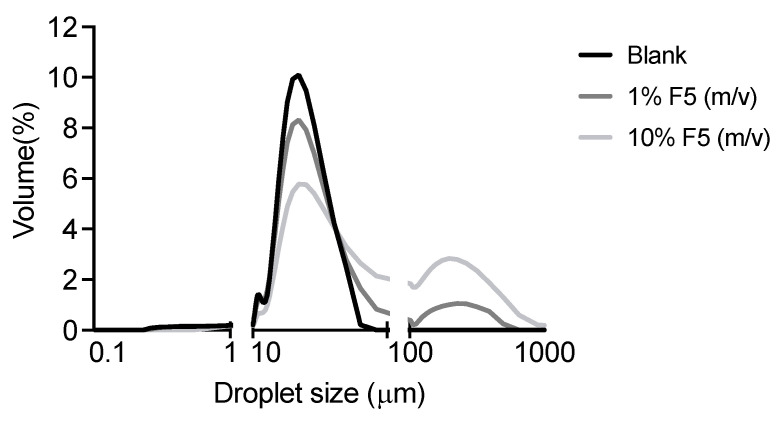
Droplet size distribution of different emulsions conducted with *Gelidium corneum* aqueous fraction (F5).

Representative micrographs of emulsions are shown in Figure 7. In bright light, all oil droplets are generally small, with emulsions containing 1% and 10% extracts presenting slightly bigger droplets than blank (Figure 8). Emulsion stability is strongly affected by the size of the dispersed phase [30,31]. 

The presence of aqueous *G. corneum* extract influences the droplet size of the emulsions. New droplets appear in the emulsion at 1%, and the size between 10 μm and 100 μm slightly decreases. This phenomenon is even more noticeable in 10% emulsion. This sudden change can be due to the extract’s interaction with the ingredients present in the emulsion, or it can be mostly influenced by the extract’s own particle size. Higher particle sizes could lead to a destabilization of the emulsion [30]. This is consistent with what happened in the centrifugation cycle with the 10% emulsion oil phase. 

##### Rheological Characterization

**Figure 9:**
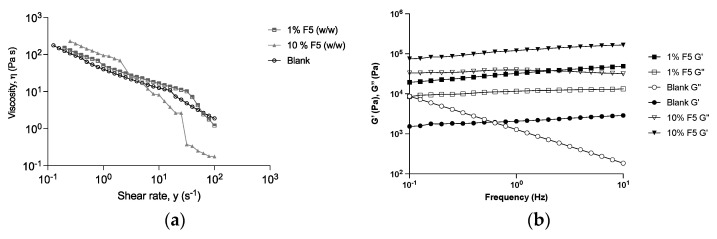
Rheology assessment of emulsions containing fraction F5 *Gelidium corneum.* (**a**) Viscosity *versus* the shear rate of different emulsions conducted with F5. (**b**) Elastic module (G′) and viscous module (G″) as a function of the frequency of the emulsions conducted with F5.

The rheological characterization of emulsions containing fraction F5 from *Gelidium corneum* was assessed (Figure 9). The viscosity of an emulsion is significantly influenced by the shear rate. This effect is attributed to the concentration of the dispersed phase. This behavior is a result of droplet crowding or structural viscosity. The viscosity vs. shear rate of the different emulsions is shown in Figure 9a: at 10^−1^ s^−1,^ the viscosity of the emulsions is higher when the percentage of F5 increases. At higher shear rates, the viscosity of the 10% F5 drastically decreases, suggesting a break in the formulation. This could imply that the water–oil interface also changed strongly [32]. The shear stress can be increased up to a specific value without any deformation taking place because the resistance is too high. If the maximum value is exceeded, the substance begins to flow [33]. After the maximum shear stress is exceeded, a sharp decrease in viscosity takes place. Therefore, the maximum shear stress for 10% F5 is lower than for the blank and 1% F5, compromising the stability of the formulation. Further tests should be conducted at different temperatures on day 0 and at different periods. This is because the assessment was preliminary and performed only at 25 °C after 7 days, only allowing an indication of stability. An assessment of the elastic and viscous modules of all samples was also performed (Figure 9b). 

All the samples showed typical solid-like behavior, with a storage modulus G′ higher than G″ (loss modulus) in frequency sweep tests within the investigated frequency range (Figure 9b). A simple way to summarize and compare the rheological characteristics of all is to evaluate the complex modulus G* and the phase angle δ (ratio between G′ and G″). The damping factor varies from 0 to infinity: the closer the value is to 0, the more elastic the material, while the closer the value is to infinity, the more the material has a viscous behavior [34]. The samples showed an increase in the δ with the presence of F5 in the emulsion, making its behavior more viscous. This result is consistent with the presence of polysaccharides in F5 since these molecules are widely used as thickening agents [35].

### 3.5. In Vivo Compatibility, Acceptability, and Antioxidant Efficacy

The in vivo efficacy test is intended to check the compatibility and acceptability of the cream and to assess its antioxidant efficacy after application under normal conditions of use. Regarding the acceptability and compatibility study, it was observed that the application of both the control blank cream and the cream containing 1% of *G. corneum* aqueous fraction did not induce any adverse reactions on the skin in any case. Thus, the formulations showed, during the study, excellent compatibility and acceptability for the skin.

**Figure 10:**
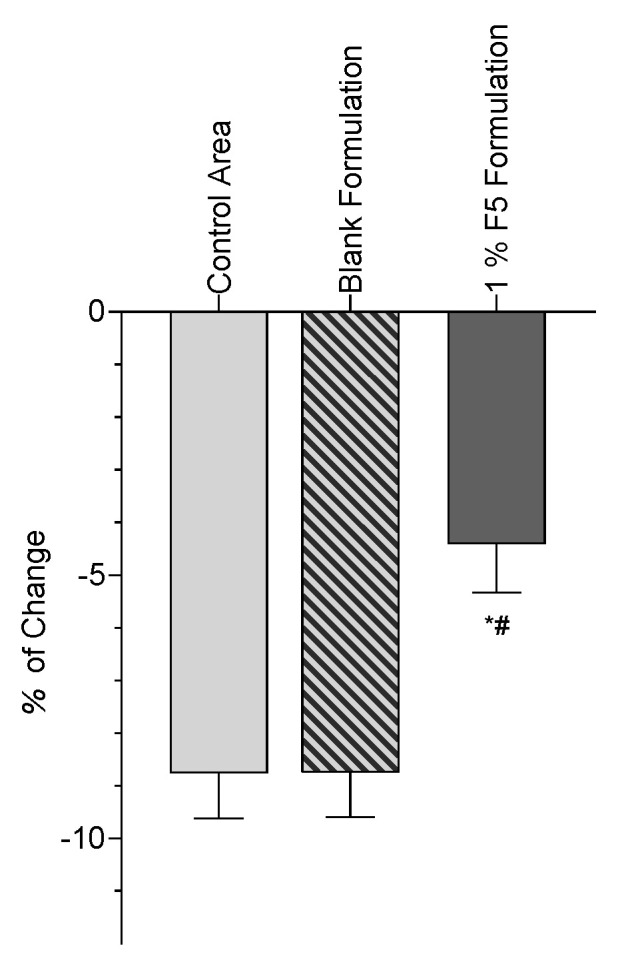
Effects of the application of the different creams on b* parameter changes before and after UVA irradiation—mean values of all volunteers (*n* = 10). The untreated area was used as a control, and a blank skin area (the control area) was also used as a negative control. The statistical comparison between products and controls is also shown, with the * and # symbols indicating statistical differences with the negative controls.

To evaluate the true changes in the b* parameter, a relative transformation in relation to D1 before irradiation was performed, and the results are displayed in Figure 10.

The color variation (b* parameter) in the control area (8.74%) is significantly higher than the variation in the treated area with the cream sample containing 1% of *G. corneum* aqueous fraction (4.39%). The difference between the variations is 4.35% one hour after UV irradiation (immediate effect). This result shows that the cream sample containing 1% of *G. corneum* aqueous fraction 1 h after UV irradiation was able to decrease the oxidation of β-carotene under UVA irradiation.

## 4. Discussion

The red seaweed *G. corneum* is harvested seasonally in Portugal and is generally associated with agar extraction. However, besides agar, other highly valuable ingredients are biosynthesized by this seaweed. Aiming to obtain enriched fractions of different chemical components, a polarity gradient extraction was performed as previously described [21]. Since this work aimed to investigate the cosmetic potential of such fractions, the first step was to assess their safety through the evaluation of in vitro cytotoxicity and inflammatory effects.

Except for the diethyl ether fraction (F3), which became cytotoxic only at 100 μg/mL, and the less polar fraction (F2), which became cytotoxic at 600 μg/mL, no cytotoxic effects were observed in HaCaT cells. Additionally, at non-cytotoxic concentrations, none of the fractions induced inflammation in RAW 264.7 cells. Inflammation and other skin impairments can be associated with excessive production of reactive oxygen species (ROS). Therefore, the incorporation of antioxidant ingredients into topical formulations brings additional advantages to human skin health.

When looking to understand the antioxidant effect of the different fractions, phenolic compounds, pigments, sulfated polysaccharides, and MAAs biosynthesized by *Gelidium* species may act as relevant antioxidant ingredients. The way these compounds neutralize free radicals will depend on their relative concentrations in the sample matrix and can act synergistically, additively, or antagonistically to inhibit reactive species [36]. Signals of phenolic compounds were detected in the NMR spectra of the F3 and F4 fractions of *G. corneum* [21]. Nevertheless, F2 also presented a relevant antioxidant capacity but a relatively low phenolic content. In fact, intense signals of more lipophilic compounds were observed in its NMR spectra [21]. This suggests that other ingredients, such as pigments and vitamins, may act synergistically as antioxidants with other molecules commonly found in seaweed species [37].

The total phenolic content (TPC) was evaluated in *G. corneum* fractions, with F4 exhibiting the highest TPC (32.92 ± 6.29 mg GAE/g extract), followed by F3 (12.80 ± 2.28 mg GAE/g extract). Regarding the DPPH radical scavenging activity, fractions F1 and F5 did not display a high reduction capacity, which is consistent with the result reported by Cavaco et al. [38] with a maximum of 10.89% of DPPH inhibition in aqueous extracts from the same seaweed collected off the Portuguese shore. Matos et al. [39], also working with the same species, reported a low scavenging capacity, with an average of 6.8 ± 1.2% DPPH inhibition for ethanolic extracts and none for the aqueous ones. However, it is worth noting that although F1 and F5 from *G. corneum* have revealed low scavenging potential, the results obtained here are still higher than those previously reported, being 15.56 ± 4.24% and 19.82 ± 2.71%, respectively. None of the tested fractions exhibited a relevant potential to reduce ferric ions in the FRAP assay compared to the commercial antioxidant BHT. On the contrary, concerning the ORAC assay, almost all fractions displayed a higher antioxidant capacity than BHT (136.4 ± 9.09 μmol TE/g extract), with F4 exhibiting the most prominent result with 3060.00 ± 222.20 μmol TE/g of extract. Unfortunately, there are no relevant studies focused on this assay on other *Gelidium* species, except those referred by Rengasamy et al. [40], which reported a value of 9.70 ± 0.38 μmol TE/g extract on a *Gelidium foliaceum* methanolic extract. Although a high phenolic content is largely correlated with relevant antioxidant activity, other compounds biosynthesized by red algae, e.g., mycosporine-like amino acids, phycobiliproteins, carotenoids, sulfated polysaccharides, etc., are reported for their antioxidant and photoprotective activities [15,17,18,19,20]. The preliminary chemical screening by ^1^H NMR and UV–Vis spectroscopy has evidenced the presence of such a group of compounds in *G. corneum* fractions, which is a starting point for a deeper chemical characterization through complementary powerful techniques such as LC–MS/MS^n^ and 2D-NMR.

Human skin responds to ultraviolet (UV) radiation by activating different pathways to protect healthy cells whenever possible. UV radiation stimulates the production of ROS that cause destructive oxidative stress, activate the arachidonic acid pathway, and mediate inflammatory responses [5]. Acute UV radiation exposure can cause sunburn and abnormal pigmentation, while long-term exposure can lead to malignant tumor development [6]. Therefore, incorporating photoprotective filters into cosmetics can make them more functional and increase the benefits offered to consumers by counteracting photoaging. Although seaweeds biosynthesize a panoply of compounds of interest, a particular group linked with UV protection is the mycosporine-like amino acids, which seem to be present only in red seaweeds [41,42]. The phenolic content evaluated in this study seems not to be related to photoprotective potential since the hydroethanolic fraction F1 had the lowest TPC level of all extracts, and the highest phenolic content fractions had no statistical differences in relation to the control. F1 could protect cells from oxidative stress through the presence of MAAs since these molecules are water-soluble. Therefore, they can be present either in the hydroethanolic fraction F1 and/or in the aqueous fraction F5, as supported by the UV and ^1^H NMR spectra. It is worth noting that the cells were not exposed to UV radiation in the presence of the different extracts to assess possible UV absorption. The radiation was instead used as a cellular stressor to evaluate if a previous extract incubation could defer the ROS that normally occurs after solar exposure. Both the F1 and F2 fractions also contain pigments in their composition. Carotenoids, for example, are lipid-soluble pigments that seem to be able to provide photoprotection against UV light-induced photooxidation in the skin, and marine algae contain up to 0.2% of carotenoids [43]. An extract obtained from *Gelidium* sp. (Alga-Gorria^®^, Laboratoires de Biarritz, France) [15] is claimed to be a powerful antioxidant with the capacity to neutralize a broad spectrum of free radicals and to prevent premature skin aging, probably due to its content in MAAs and carotenoids. Moreover, the species *Gelidium amansii* was shown to produce phycocyanins and phycoerythrins. According to Sukwong et al. [44], these valuable water-soluble pigments are also associated with skin protection against aging.

The healing effect observed with the aqueous fraction F5 can be due to the presence of polysaccharides and/or MAAs in this fraction [21]. Effectively, several studies note the healing ability of different polysaccharides found in macroalgae [45,46,47,48], such as ulvans (green algae), galactans (red algae), and fucoidans (brown algae). Fraction F2 from *G. corneum* also had a relevant healing action. This could be due to lipids and/or pigments [21], since these molecules are also used in cosmetic formulations to help afflicted skin [44].

One of the main challenges in formulating a new cosmetic product is its sensorial aspect. Therefore, natural bioactive ingredients would require some adaptations to be successfully incorporated into novel formulations. When looking at skin diseases, the impact of cosmetics is somewhat limited. However, conditions caused by barrier defects can be addressed with cosmetic use. These are largely caused by trans-epidermal water loss [48,49], and moisturizing formulations might help address such problems [50,51]. Marques et al. [28] have created a topical formulation with extracts from the plant *Cynara scolymus*. This previously optimized O/W emulsion was adapted for this work due to the benefits offered by O/W formulations, such as quicker skin absorption and avoiding any greasy feeling post-application. The most hydrophilic fraction (F5) exhibited a good extraction yield, did not impact skin microbiota [21], and was chosen to prepare the cosmetic formulation reported here. It also takes advantage of the thickening and gelling properties of polysaccharides, its main components. These large molecules may also have an impact on the particle size distribution within the formulation since the more extract present, the higher the percentage of particles bigger than 100 μm. Polysaccharides are known for their healing and water retention abilities and are already used in the medical and cosmetic fields for that specific reason [52]. Agar extracted from *G. corneum* is also used as a thickener [53]. This effect was seen in the formulation containing the highest concentration of extract and is consistent with the swelling capacity observed in the polysaccharide structure once it is in contact with water [54]. The in vivo studies assessed with the emulsion containing an extract from *G. corneum* demonstrated its antioxidant capacity through its ability to decrease the oxidation of β-carotene when exposed to UVA radiation. In addition, no negative side effects were observed in human volunteers.

## 5. Conclusions

The incorporation of *G. corneum* aqueous extract (1%) into the developed cosmetic formulation has improved its antioxidant capacity, as evidenced by in vivo assays. The choice of this extract was influenced by its in vitro healing capacity, great sensorial aspect (caramel color, no strong smell), and ability to properly disperse in a formulation with high water content. No adverse effects were observed in human volunteers, highlighting the potential of this marine resource for multitarget dermatological applications able to prevent oxidative impairments that affect the equilibrium of healthy skin. However, more in-depth studies are needed in the future concerning the long-term effects and mechanisms of action against UV damage promoted by ingredients extracted from *G. corneum* collected off the Portuguese shore.

## Figures and Tables

**Table 1:** Composition of the prepared emulsions.

Phases	Ingredients (INCI)	O/W Emulsion Quantitative Composition (%, *w*/*w*)		
		Blank Emulsion	Emulsion 1%	Emulsion 10%
Oily Phase	Polyglyceryl-3 Dicitrate/Stearate	3.00	3.00	3.00
	Cetearyl Alcohol	7.00	7.00	7.00
	Liquid Paraffin	2.50	2.50	2.50
	Decyl Oleate	4.50	4.50	4.50
	Almond Oil	5.00	5.00	5.00
Aqueous Phase	Glycerin	5.00	5.00	5.00
	Aqua	72.80	71.80	62.80
	Methylparaben	0.18	0.18	0.18
	Propylparaben	0.02	0.02	0.02
Added Extracts	*Gelidium corneum* aqueous extract	-	1.00	10.00

**Table 2:** Antioxidant capacity of *Gelidium corneum* fractions. Values represent the mean ± standard error of the mean (SEM) of three independent experiments conducted in triplicate.

	Assay	TPC	DPPH	FRAP	ORAC
Fraction		(mg GAE/g Extract)	(EC_50_; μg/mL)	(μM FeSO_4_/g Extract)	(μmol Trolox/g Extract)
F1		4.37 ± 1.54	>1000.00	31.13 ± 1.31	46.80 ± 1.17
F2		10.64 ± 1.19	991.60 (875.80–1127.00)	19.22 ± 2.46	2868.00 ± 72.29
F3		12.80 ± 2.28	399.60 (338.10–471.80)	49.02 ± 5.27	2916.00 ± 132.80
F4		32.92 ± 6.29	973.10 (842.90–1128.00)	19.11 ± 2.54	3060.00 ± 222.20
F5		3.63 ± 0.29	>1000.00	27.96 ± 3.10	57.58 ± 4.26
BHT		--------------	184.70 (162.20–210.50)	1948.00 ± 239.10	136.4 ± 9.09

## Data Availability

The data presented in this study are available in the article.
